# Blood Lactate Concentration Is Not Related to the Increase in Cardiorespiratory Fitness Induced by High Intensity Interval Training

**DOI:** 10.3390/ijerph16162845

**Published:** 2019-08-09

**Authors:** Todd A. Astorino, Jamie L. DeRevere, Theodore Anderson, Erin Kellogg, Patrick Holstrom, Sebastian Ring, Nicholas Ghaseb

**Affiliations:** Department of Kinesiology, California State University—San Marcos, San Marcos, CA 92096, USA

**Keywords:** high intensity exercise, blood lactate concentration, maximal oxygen uptake, individual responsiveness to training

## Abstract

Background: There is individual responsiveness to exercise training as not all individuals experience increases in maximal oxygen uptake (VO_2_max), which does not benefit health status considering the association between VO_2_max and mortality. Approximately 50% of the training response is genetic, with the other 50% accounted for by variations in dietary intake, sleep, recovery, and the metabolic stress of training. This study examined if the blood lactate (BLa) response to high intensity interval training (HIIT) as well as habitual dietary intake and sleep duration are associated with the resultant change in VO_2_max (ΔVO_2_max). Methods: Fourteen individuals (age and VO_2_max = 27 ± 8 years and 38 ± 4 mL/kg/min, respectively) performed nine sessions of HIIT at 130% ventilatory threshold. BLa was measured during the first and last session of training. In addition, sleep duration and energy intake were assessed. Results: Data showed that VO_2_max increased with HIIT (*p* = 0.007). No associations occurred between ΔVO_2_max and BLa (r = 0.44, *p* = 0.10), energy intake (r = 0.38, *p* = 0.18), or sleep duration (r = 0.14, *p* = 0.62). However, there was a significant association between training heart rate (HR) and ΔVO_2_max (r = 0.62, *p* = 0.02). Conclusions: When HIIT is prescribed according to a metabolic threshold, energy intake, sleep status, and BLa do not predict ΔVO_2_max, yet the HR response to training is associated with the ΔVO_2_max.

## 1. Introduction

One adaptation to moderate intensity continuous training (MICT) is a significant increase in maximal oxygen uptake (VO_2_max) [1], which reduces mortality risk [2]. It is apparent that some individuals reveal marked increases in VO_2_max in response to training, whereas, others experience little to no change [3]. Approximately 50% of the change in VO_2_max (ΔVO_2_max) with training is genetic [3], with the other 50% due to variations in habitual dietary intake, sleep status, physical activity, and the specifics of the training regime [4]. Ross et al. [5] showed greater increases in VO_2_max in response to high amount, higher intensity (75% VO_2_max) versus lower intensity MICT (50% VO_2_max). This suggests that vigorous continuous exercise may optimize ΔVO_2_max, which seems important as a 1 metabolic equivalent (MET) increase in VO_2_max is associated with a 19% reduction in all-cause mortality [6].

One additional factor that may mediate training responsiveness is the absolute metabolic stress of training [4]. Although there are various ways to monitor this, one approach is via the measurement of blood lactate concentration (BLa). Previous studies show that this measure may serve as an index of intramuscular stress [7] and may be related to the initiation of signaling pathways that regulate muscle plasticity in response to training [8]. It is apparent that the accumulation of BLa follows a threshold response in that, at intensities below the work rate coincident with the lactate threshold, there is little change in BLa, whereas, at work rates above the lactate threshold, BLa increases dramatically due to the enhanced recruitment of higher threshold motor units and greater activation of glycolysis [9]. In three groups of men (VO_2_max = 46–71 mL/kg/min) of similar aerobic fitness, Scharhag-Rosenberger et al. [10] showed dissimilar increases in blood lactate concentration (BLa) during prolonged cycling at 60% and 75% VO_2_max, which suggests discrepant metabolic strain at identical relative intensities. In addition, these authors reported that some of their participants were unable to complete the prolonged exercise bouts, which was partially explained by the onset of neuromuscular fatigue or enhanced fast-twitch motor unit recruitment, which likely varied across subjects. Recently, the BLa response to moderate intensity continuous exercise at 65 percent of peak power output (% PPO) was significantly associated with ΔVO_2_max seen with chronic training [11], which suggests that the metabolic response to acute exercise may be predictive of the adaptive response. Whether this relationship also occurs in response to high intensity interval training (HIIT) is unknown. Elucidating predictors of ΔVO_2_max with chronic exercise such as HIIT is important considering that this modality elicits superior increases in VO_2_max than MICT [12]. Moreover, VO_2_max is related to mortality [6], and better understanding predictors of the VO_2_max response to training may help clinicians better utilize physical activity when employing exercise-based rehabilitation.

Health and fitness practitioners implement exercise training in their clientele to promote gains in fitness and body composition which likely lead to improved health status. In addition, it is likely that they try and identify specific variables that impact training responsiveness to optimize exercise programming for their clientele. In this preliminary study, we measured changes in BLa during a brief HIIT regime to determine if the adaptive response is predicted by BLa changes during training. Participants’ sleep status and dietary intake were also measured, as Hawley et al. [13] and Samuels et al. [14] suggest that they are related to training responsiveness. In the case of sleep deprivation, it is associated with fatigue and may make individuals more susceptible to overtraining [13]. In addition, Mann et al. [4] postulated that the effect of habitual dietary intake on resultant variations in the training response is unknown. Overall, it is plausible that adequate energy intake as well as sleep serve to promote recovery from individual sessions, which in turn may benefit the resultant adaptation to training. It is hypothesized that the BLa response to training is significantly related to ΔVO_2_max.

## 2. Materials and Methods

Participants: Three men and eleven women (age and VO_2_max = 27 ± 8 years and 38 ± 4 mL/kg/min, respectively) who perform >150 min/week of exercise in the last 12 months, including resistance training, aerobic exercise, noncompetitive sport, and group exercise, participated in this study. They were recruited via convenience sampling. They completed a standard health-history questionnaire, which contained information pertaining to their current physical activity regimen. Participants also provided written informed consent to take part in the study, whose procedures were approved by the University Institutional Review Board. The study was carried out in accordance with the rules of the Declaration of Helsinki.

Design: VO_2_max was assessed before and after nine sessions of HIIT. On days 1 and 9 of training, BLa was measured. Changes in sleep status and dietary intake were monitored during the study. Data concerning ΔVO_2_max and ventilatory threshold in response to this regimen were previously reported [15]. The present study summarized changes in these outcomes (BLa, sleep status, and dietary intake) that were only obtained from the experimental group who underwent interval training. Sessions occurred at the same time of day within participants, were preceded by a 24 h abstention from physical activity, and were separated by ≥24 h. Participants were instructed to record habitual physical activity during the study in a written log, and were instructed to maintain this behavior during the study.

Testing of maximal oxygen uptake: Participants performed incremental exercise on an electrically braked cycle ergometer (Velotron Dynafit Pro, RacerMate, Seattle, WA, USA). After a 2 min warm-up at 40–70 Watt (W), the work rate was increased by 20–35 W/min until volitional exhaustion, represented by cadence <50 rev/min. After 10 min of pedaling at 10% PPO, participants pedaled at 105% PPO to volitional exhaustion [16]. VO_2_max was identified as the average of these two values. VO_2_max testing was repeated at least 48 h after the last training session. During all bouts, HR was measured continuously using telemetry (Polar Electro, Beth Page, NY, USA), and gas exchange data were acquired every 15 s using indirect calorimetry (ParvoMedics True One, Sandy, UT, USA).

Assessment of blood lactate concentration, sleep status, and dietary intake: During sessions 1 and 9 of HIIT, BLa was determined using a portable monitor (Lactate Plus, Nova Biomedical, Waltham, MA, USA) and lancet (Owen Mumford, Inc., Marietta, GA, USA). After a 5 min rest, the fingertip was washed with a damp paper towel and dried, and BLa was measured by using the second drop of blood, as the first one was wiped away. This procedure was repeated immediately after intervals 4 and 8. The change in BLa during the session of HIIT was calculated as the difference between the pre-exercise and interval 8 values. In addition, the change in BLa in response to training was calculated as the difference in the sum of BLa recorded after intervals 4 and 8 on day 9 versus day 1 of training. On day 1, participants recorded their dietary intake in the 24 h prior and replicated this prior to session 9 to standardize the fed state before these sessions.

Dietary intake was measured for three days prior to and for three days during the last week of training. Participants recorded all food and drink ingested over this period in a log. This information was analyzed using software (http://ndb.nal.usda.gov/ndb/foods/list) to determine energy intake (in kilocalories). Sleep status was quantified every day of training, as participants reported the number of hours they slept on the night prior, and an average value was calculated.

High intensity interval training: At least 48 h after baseline testing, participants initiated HIIT at a work rate equal to 130% ventilatory threshold (VT), which was equal to 70% PPO in our participants. Ventilatory threshold was estimated by two experienced reviewers independently using the methods of Caiozzo et al. [17]. If their evaluation differed, consensus was reached by consulting a third investigator. Sessions were held three days/week for three weeks and were performed on the same cycle ergometer. Subjects performed eight 1 min intervals on days 1–3 of training, nine on days 4–6, and ten on days 7–9 [18]. Sessions were preceded by a 5 min warm-up at 10% PPO, and intervals were separated by a 75 s active recovery at 10% PPO. Heart rate (HR) was measured continuously using telemetry (Polar Electro USA, Beth Page, NY, USA). The HR response to training was represented by the average HR attained at the end of each interval across all sessions of training.

Data analyses: Data were expressed as mean ± standard deviation (SD) and were analyzed using SPSS Version 24.0 (IBM, Armonk, NY, USA). The Shapiro–Wilk test was used to assess normality. A two-way analysis of variance (training = pre versus post, time = three levels) with repeated measures was performed to identify differences in BLa. The Greenhouse–Geisser correction was used to account for the sphericity assumption of unequal variances across groups. If a significant F ratio occurred, Tukey’s post hoc test was used. Pearson’s pairwise correlation was used to determine relationships between variables. Statistical significance was set as *p* < 0.05.

## 3. Results

Training fidelity: Training elicited 90% ± 5% PPO (79%–96% across participants), which verifies the intensity of this regime. VO_2_max was increased by 6% with HIIT, and this change was equal to 0.15 ± 0.13 L/min (range = −0.06–0.47 L/min) and 2.4 ± 1.8 mL/kg/min (range = −0.9–4.7 mL/kg/min) across participants.

Changes in blood lactate concentration in response to training: BLa increased during HIIT (*p* < 0.001) yet there was no difference from day 1 to 9 of HIIT (*p* = 0.91) or trainingXtime interaction (*p* = 0.87). The change in BLa during session 1 (10.5 ± 2.2 mM) did not differ compared to session 9 (10.3 ± 2.2 mM). The overall change in BLa from pre- to post-training was equal to −0.36 ± 2.4 mM. Four participants showed a greater than 1 mM reduction in BLa from pre- to post-training, whereas, five participants showed increases in BLa above 1 mM.

Changes in sleep status and energy intake: Sleep duration was equal to 7.5 ± 0.7 h and ranged from 6.0–8.8 h per night across participants. Dietary intake did not change from pre- (2050 ± 686 kcal) to post-training (2074 ± 639 kcal) (*p* = 0.39), although it varied from 950–3400 kcal/d across participants.

**Figure 1:**
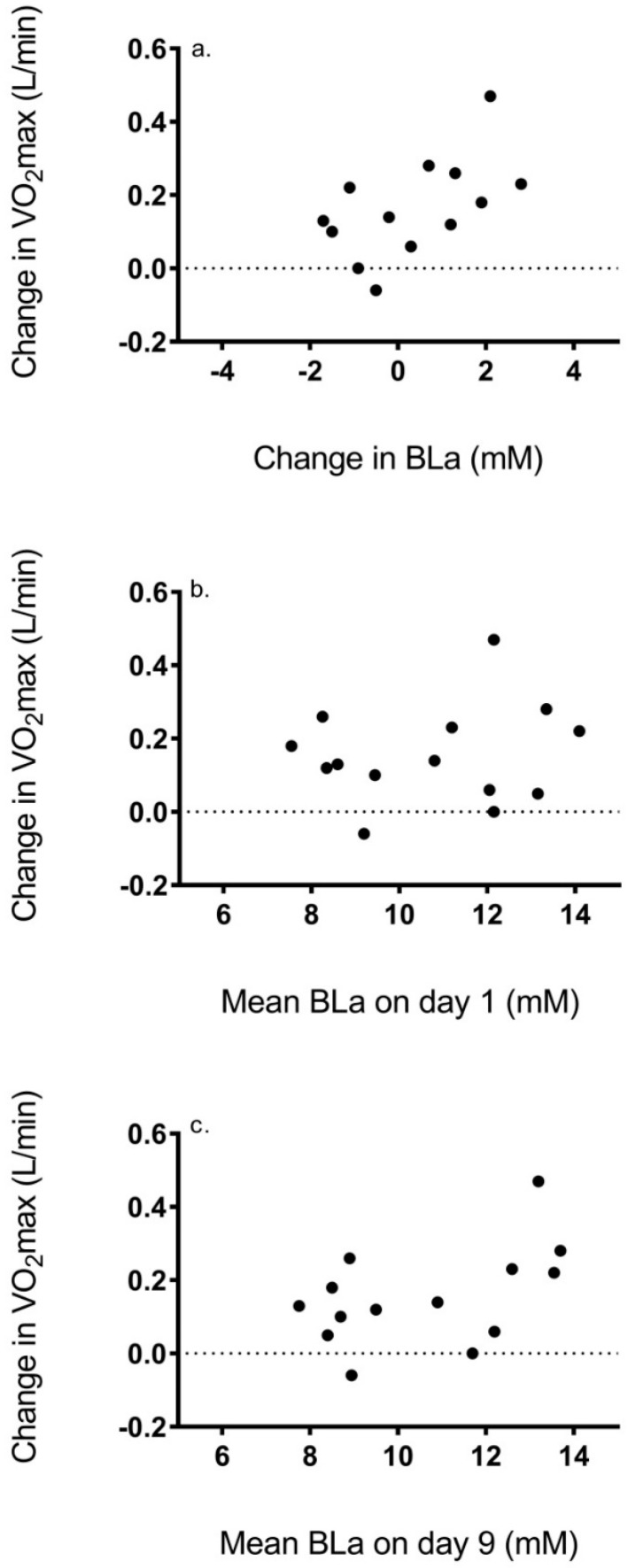
Association between change in VO_2_max and (**a**) change in blood lactate concentration, (**b**) blood lactate concentration on day 1 of training, and (**c**) blood lactate concentration on day 9 of training.

**Figure 2:**
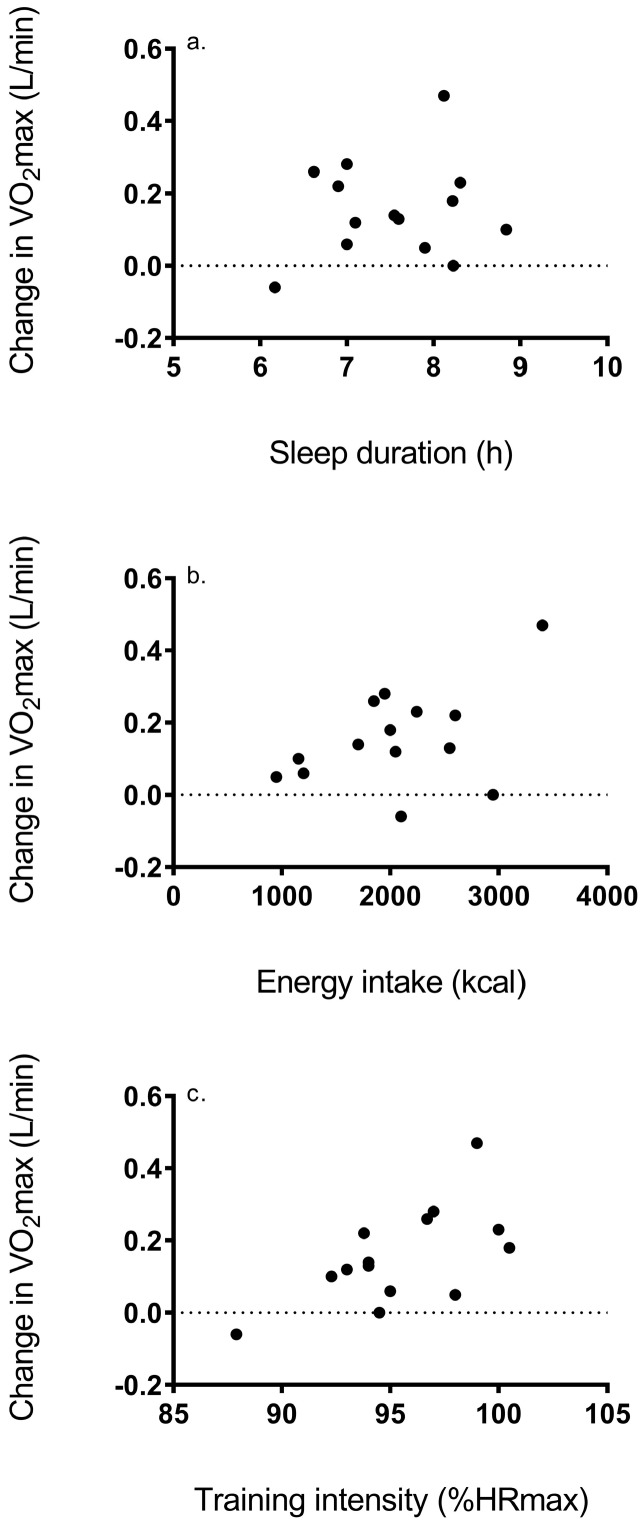
Association between change in VO_2_max and (**a**) sleep duration, (**b**) dietary intake, and (**c**) training intensity expressed as % HRmax.

Relationship between ΔVO_2_max and BLa, sleep status, and dietary intake: No correlation was shown between absolute ΔVO_2_max and the change in BLa (r = 0.44, *p* = 0.10) (Figure 1a). In addition, no correlation was shown between ΔVO_2_max and the mean BLa on day 1 (r = 0.17, *p* = 0.57) or 9 of training (r = 0.48, *p* = 0.08) (Figure 1b,c). In addition, results showed no correlation between ΔVO_2_max and sleep status (r = 0.14, *p* = 0.62) or energy intake (r = 0.38, *p* = 0.18) (Figure 2a,b).

Relationship between ΔVO_2_max and training heart rate: Heart rate in response to training ranged from 88%–100% percent of maximal heart rate (HRmax), with a mean value equal to 95% ± 3% HRmax. Results revealed a significant correlation between ΔVO_2_max and training HR (r = 0.62, *p* = 0.02) (Figure 2c).

## 4. Discussion

Previous studies document a heterogeneous BLa response to continuous exercise [10] and that the change in BLa in response to acute continuous exercise at 65% PPO predicts the resultant training response [11]. Our study examined if the BLa response to short-term HIIT is associated with ΔVO_2_max. The results oppose our hypothesis and they suggest that HR response to training is significantly associated with change in VO_2_max, yet BLa, sleep duration, and energy intake are not. These preliminary data suggest that the cardiovascular strain of HIIT prescribed according to a metabolic threshold may predict resultant responses to training.

Our data showing no correlation between ΔVO_2_max and the BLa response to HIIT refute previous results [11]. However, there are methodological differences between studies which may explain these discrepancies. First, our sample contained men and women, while the former study included only men. It is apparent that muscle fiber type may differ between men and women [19], leading to greater reliance on glycolysis and resultant blood lactate accumulation in men who have a greater proportion of higher threshold motor units. Moreover, men exhibit higher BLa in response to HIIT and sprint interval training (SIT) versus women, which may be due to the greater work completed during training due to a propensity to maintain a higher cadence, especially during SIT [20]. Second, HIIT was performed at an intensity equal to 90% PPO, whereas, in the former study, training was performed at 65% PPO. This different composition of training led to markedly different BLa responses, as our subjects exhibited BLa ranging from 7.6–14.1 mM, which was higher than values exhibited in their study (6–8 mM). Lastly, our regimen was prescribed according to VT, which may potentially reduce variability in metabolic stress versus their regimen, which may have had select participants training above or below the workload associated with VT. Further work is merited to examine if the BLa response to HIIT prescribed according to an absolute intensity is associated with ΔVO_2_max.

Our data showed no association between ΔVO_2_max and sleep duration or calorie intake. Sleep deprivation may mitigate the adaptive response to training by eliciting fatigue, which may reduce performance [21]. Previous results exhibit that one (25–30 h of sleeplessness) [22] and three nights of sleep deprivation (3 h of sleep per night) [23] decreased time to exhaustion and muscular strength in athletes, whereas, in basketball players, two additional hours of sleep per night for up to seven weeks were consequent with increased speed and shooting performance [24]. Our data show that the participant with the lowest amount of sleep (6.0 h) did not exhibit increases in VO_2_max with HIIT. However, two additional participants who received 8 h per night of sleep also showed minimal increases in VO_2_max, which suggests that sleep duration by itself may not predict training responsiveness. Two of these participants also completed 7 h/week of physical activity outside the HIIT regimen, so an effect of overreaching on their lack of response may exist. Our participants’ habitual dietary intake ranged from 1.8–3.8 kcal/kg body mass. Two men and two women exhibiting substantial increases in VO_2_max (0.13–0.47 L/min) revealed dietary intakes approaching 3.8 kcal/kg body mass, whereas, two participants ingesting less than 2 kcal/kg revealed no change in VO_2_max in response to training. These individual results reveal that nutritional state may impact the training response. Nevertheless, in adults with diabetes, the addition of post-exercise protein did not affect adaptation to training [25], which may indicate that diet has little impact on training responsiveness. 

A significant association between HR response to training and ΔVO_2_max was revealed (Figure 2). Our results reveal that participants with the greatest increases in VO_2_max exhibited training HR above 95% HRmax. Whether this greater adaptation is due to some unique sympathetic response or alternatively, maintaining a higher cadence during HIIT, which would elicit greater work, is unknown. In sedentary men, six sessions of HIIT increased VO_2_max and PPO [26], with these outcomes associated with the ratio of low to high frequency power of R-R oscillation, which represents sympathovagal balance. Higher vagal activity has been shown to be directly related to ΔVO_2_max in response to MICT [27], and further study is merited to confirm this result in response to other HIIT regimens.

One limitation of our study is that dietary intake was quantified through self-reported logs, which are prone to underreporting [28]. Studies show that the pre-exercise nutritional state may alter the molecular response to training [13], so monitoring participants’ dietary patterns before and after each session may be useful to better understand the effects of nutrition on training responsiveness. Sleep status was assessed by identifying the duration of sleep the night before each session, rather than using various questionnaires, which may be more valid to assess sleep quality [29]. Thus, the reliability of our relatively simple measure is unknown. In addition, our sample included both men and women who may show unique responses to interval training [30], yet our study was underpowered to examine this potential effect of sex. No measure of critical power was performed in our study, so it is likely that participants were training at different workloads within the heavy and/or severe intensity domain. Lastly, our training protocol was relatively brief, so data cannot be used to explain responsiveness to prolonged regimens of interval training.

## 5. Conclusions

Overall, training HR was associated with the VO_2_max response to HIIT, yet dietary intake, sleep duration, and BLa accumulation were not predictive of this response. Overall, these preliminary data suggest that the absolute cardiovascular strain may be a mediator of the adaptive response to interval training.
